# “This was never about a virus”: Perceptions of Vaccination Hazards and Pandemic Risk in #Covid19NZ Tweets

**DOI:** 10.1007/s10912-024-09859-9

**Published:** 2024-07-10

**Authors:** Maebh Long, Andreea Calude, Jessie Burnette

**Affiliations:** 1 English Programme, University of Waikato, Private Bag 3105, Hamilton, 3240 New Zealand; 2 University of Waikato, Hamilton, New Zealand

**Keywords:** World Risk Society, Vaccination, COVID-19, Twitter (X), Hashtags, Aotearoa New Zealand

## Abstract

In this paper, we draw on qualitative methods from the medical humanities and quantitative approaches from corpus linguistics to assess the different mappings of pandemic risks by Twitter (X) users employing the #Covid19nz hashtag. We look specifically at their responses to government measures around vaccines between August and November 2021. Risk, we reveal, was a major discursive thread in tweets during this period, but within our tweets, it was the vaccine rather than the virus around which hazard perception and response were grouped. We find that the discursive stance of those opposed to the vaccine evoked entangled medical and political hazards, untrustworthy experts, obscure information, restrictions on sovereignty, threats to children, and uncertain future dangers, all of which positioned them within what Ulrich Beck termed the world risk society. We also found that these narratives of risk manifested in specific Twitter styles, which employed a consistently larger number of hashtags. The lack of conjunctions between the hashtags, we argue, encouraged a disordered reading of doubt and precaution, as the hashtags presented triggering phrases whose interconnections were hinted at rather than specified. By contrast, those who tweeted in support of government measures were rhetorically led by solutions rather than risks, with one exception: their perception of those who were vaccine opposed. We use scholarship on risk and precautionary logic to map out the contrasting positions in tweets addressing Aotearoa New Zealand’s pandemic experience during the closing months of 2021.

## Introduction

A pandemic is always risky business. The viral or bacterial cause of a pandemic presents serious hazards to physical health, but the disease itself, as well as attempts to mitigate its impacts, also presents threats to mental health, the economy, political stability, and the environment. Vaccinations are particularly bound up in the entangled risk nexus of a pandemic. To those who see vaccinations as a vital part of healthcare, anti-vaccination positions denote deeply risky behavior stemming from a toxic mix of paranoia, arrogance, and ignorance. To those who see vaccinations as the injection of an insufficiently tested, poisonous substance into the body by profit-driven companies, pro-vaccination positions are deeply risky, stemming from naivety, blind optimism, or servile compliance to governmental decrees.

The varied perceptions of entangled risks during a pandemic can place the public in a version of modernity that Ulrich Beck has termed the “world risk society.” The world risk society is a modernity of transnational hazards arising from a complex scene of entangled causes and effects that render risk difficult to assess, in which government actions appear inadequate, and experts seem to operate at scales incompatible with public need (Beck 1992). In 2021, in a short letter to the *Canadian Journal of Public Health*, Fardin Mansouri and Fatemeh Sefidgarbaei described COVID-19 as a “disease of the risk society.” The virus, they argued, is a peril that has no boundaries and is a genuine “phenomenon of globalization.” The long-term impacts of the virus are as yet unknown, and the pandemic was marked by deep social stress, disruption, and isolation (Mansourie and Sefidgarbaei, 2021, 36). To Mansouri and Sefidgarbaei’s outline of the elements that make COVID-19 a condition of world risk, we add the specific risks and perceptions of risk that coalesce around vaccinations. Although Beck’s delineation of a world bound by intertwined threats stemmed originally from environmental issues, his research can be used to shed light on public responses to the pandemic experience of a world distinguished by hazards (Wardman and Lofstedt 2020; Constantinou 2020).

In this article, we draw on the world risk society and mappings of entangled risk to illuminate public perceptions of pandemic threat in Aotearoa New Zealand, where vaccinations were made available and then mandated relatively late in the global pandemic. We present an analysis of pandemic risks as depicted by users on the platform then called Twitter, now X, who employed the #Covid19nz hashtag between August and November 2021, looking specifically at their responses to government measures around vaccines. The period between August and November 2021, while short, was a time of significant change for Aotearoa New Zealand: vaccine rollout for the general population began (Ardern 2021), there was a nationwide return to the lockdown of Alert Level 4,^1^ a persistent Delta outbreak saw the government phase out the elimination strategy that had kept the country largely virus-free (Manatū Hauroro Ministry of Health 2022), and announcements were made about imminent mandatory vaccination requirements (Hipkins 2021).

Aotearoa New Zealand’s low COVID-19 mortality rate during the first two years of COVID outbreaks adds a unique, at times contrasting, case study to global scholarship on the pandemic, vaccinations, and risk perceptions. Much of this difference lies in the fact that by August 1, 2021, the country had experienced only 26 confirmed deaths caused by COVID-19, compared to 5,060 in Ireland, an island nation of similar population, and 4.24 million deaths worldwide (Our World in Data, n.d.). Confirmed case numbers were also low: by the same date, there had been only 2,518 cases in Aotearoa New Zealand, compared to 300,627 in Ireland and 198.28 million worldwide (Our World in Data, n.d.). As such, for the majority of the population in Aotearoa New Zealand, the virus was a threat still to come rather than one prevalent in the community, with illness and death predominantly occurring abroad rather than in people’s homes. However, as the vaccine was made available in Aotearoa New Zealand, it was joined by global discourses of vaccine risk. 2020 had seen small, isolated protests against lockdowns, but during this period, objection to lockdowns and vaccine mandates grew into a vocal minority, with 2,000 people protesting outside parliament on November 9, 2021 (Beattie and Priestley 2021; McClure 2021).

As the public worked through the challenges of the pandemic, they took to social media to discuss the potential impacts of the virus, further lockdowns, vaccinations, and the vaccine mandate. In this paper, we reveal that risk was a major discursive thread in tweets during this period but that, within our tweets, it was not the virus that created the conditions for uneven perceptions of hazards. Instead, it was the vaccine that catapulted certain users into the world risk society. We use Beck, then, to map out contrast rather than consistency in risk perception and risk depiction in Aotearoa New Zealand during the closing months of 2021. We engage with the affective implications of dwelling in the world risk society as well as its material conditions, using the concept of a risk society not to make substantive claims about the objective state of life in Aotearoa New Zealand at the end of 2021 but to unpack the various subjective experiences of risk as articulated by those discussing life during the pandemic, and its associated hazards, at this time. As such, we use Beck to examine and emphasize attitudes towards pandemic risks, particularly towards the perceived risks and benefits associated with the vaccine.

As we show, those who took positions against the vaccine painted scenes in which entangled medical and political hazards, untrustworthy experts, obscure information, restrictions on sovereignty, threats to children, and emphasis on uncertain future dangers abounded. We found that these users consistently used larger numbers of hashtags, which served to emphasize messages of threat and amplify their tweets but also encouraged a disordered reading of doubt and precaution, as they presented triggering phrases whose interconnections were hinted at rather than specified. These users’ tweets drew on, and exemplified, the risk responses of a world risk society.

Against these discourses of threat were the tweets of those in support of government measures, for whom the lockdowns and vaccinations were valued solutions to pandemic hazards, as they were ways of resolving risk that were supported by science, backed by a government they considered relatively well-intentioned, and enforced by mandate seen as reasonable within the pandemic context. If those opposed to the vaccine presented a complex world of unpredictable risk, those in support presented a world of ready individual and community-based solutions. There was one main exception, however: the threat posed by the vaccine-hesitant and those they perceived as anti-vaccinators, who were represented as unpredictable hazards, thereby displacing the pandemic risk from viral to human form.^2^ So, in our data, risk during the pandemic resides either in other people, or other people and the vaccine, but rarely in the virus itself.

The research team for this project comprises humanities and social science scholars whose approach blended the qualitative method of textual analysis common in the medical humanities and English literature with the quantitative method of statistical analysis regularly used within corpus linguistics. Following sections on background, methodology, and results, we provide five discussion sections and a conclusion. The initial three sections—background, methodology, and results—broadly conform to modes of presentation common within the social sciences, although they add attention to, and quotation of, the precise wording of secondary material more common to the modes of analysis found in literary studies. The discussion sections, whose analysis primarily aligns with literary studies-influenced approaches within the critical medical humanities (Whitehead and Woods 2016; Bolaki 2016), bring together close readings of the tweets, Beck’s risk theories, and wider interpretation of scholarly literature, as well as incorporating statistical results gleaned from our coding. The result is an article that is neither strictly of the social sciences nor of the humanities but an amalgamation of both, thereby furthering the medical humanities’ interdisciplinary potential.

## Background

In Beck’s (1992) representation of modernity, the confidence of the late nineteenth and early twentieth centuries in science and technology’s ability to decrease risk has, in contemporary society, given way to an understanding of risk as caused and escalated by progress itself. The hazards confronting the public are not “natural” hazards but result from modernization, such that unintended consequences of modern life are a dominant social force. Nor, for Beck, do these risks trickle simply from the powerful to the marginalized: hazards move across social classes and national borders to create impossibly complex global entanglements. It is not, or not just, that the world has become more dangerous, but that “uncontrollable risk” has become de-bounded, that is, no longer contained by national borders (Beck 2002). The complexities of modernization mean that cause and effect must be mapped across seemingly disparate objects and events, which render risks increasingly hard to trace and predict. Hazards are produced not only by “bad” decisions or strategies but as the side effects of “good” policies and progressive change (Beck et al. 2013). More and more, the public has to focus on hazards that they cannot see with the naked eye, hazards that may not be realized during their lifetimes, and hazards that only the instruments of science can relate. While risks are too complex to be readily mapped by individuals, the responses generated by experts can often seem inappropriate at the individual level, with the result that a core aspect of the risk society is suspicion of expertise. The proliferation of global risks also generates a survival instinct that can lead to short-term, individualistic positions rather than strategies that consider long-term public good (Beck 2006). Beck’s formulation of modernity and risk has been used to reflect on the COVID-19 pandemic from a range of perspectives, including the improvements that could be made to science communication after the pandemic (Pietrocola et al. 2021), post-pandemic climate change policies (Cooper, Heath, and Nagel 2022), governmental responses to the pandemic (Giritli Nygren and Olofsson, 2020), ageism during the pandemic (Cook et al. 2021), and the pandemic and machine learning (Moss and Metcalf 2020).

In the world risk society, the public is encouraged to view the world with careful paranoia, alert to deception and indifference: “Everything must be viewed with a double gaze, and can only be correctly understood and judged through this doubling. The world of the visible must be investigated, relativized and evaluated with respect to a second reality, only existent in thought and yet concealed in the world” (Beck 1992, 72). When faced with the invisible, air-borne droplets of the virus and the visible threat of the vaccine needle, significant minorities have chosen to respond to the proliferating threats of contemporary risk society by elevating the risk of the perceptible. That is, of the vaccine. Although vaccinations are a major success in the history of medicine, paradoxically, their successes have enabled anti-vaccine lobbies to thrive: vaccines’ effective control of so many illnesses have made them seem less hazardous than the alleged reactions caused by the vaccines (Salmon et al. 2015, 392). This success makes assessing the value of each new vaccine harder, leading to vaccine hesitancy caused by the public’s difficulty in calculating the risk versus the benefit of a vaccine (Lane et al. 2018).

In Aotearoa New Zealand, the rollout of the vaccine to a population that had experienced very few community cases was also a potential factor in vaccine hesitancy and skewed risk perception, as few people had seen the disease firsthand (Trnka et al. 2021). Anxieties regarding the risks of vaccinations are long-standing, and a search of the MEDLINE database showed that in the early twenty-first century, searches using the keywords *vaccine risks* were five times more frequent than searches using *vaccine benefits* (Andre et al. 2008, 140). Social media frequently exacerbates the problem. A study in *Nature* of anti-vaccination rhetoric in 2020 analyzed 100 million individuals on Facebook and found that the higher number of anti-vaccination sites than pro-vaccination sites, combined with the entanglement of anti-vaccination and undecided positions, gives the impression that anti-vaccination positions are more socially prevalent than they are (Johnson et al. 2020, 231). In particular, anti-vaccination sites blend concerns about safety, conspiracy theories, and alternative medicines to create narratives that are potentially very attractive to a range of social media users, particularly during a pandemic (Johnson et al. 2020, 231). Social media sites, as sources of information and personal connection, operate as what Jeongwon Yang (2021, 1205) terms “amplification stations of COVID-19 risk and eventually, evoke behavioral responses.” Significantly for a discourse of risk, Yang (2021) found that the negative emotions associated with a pandemic are propagated further than positive ones.

Steven Lloyd Wilson and Charles Wiysonge’s large-scale study of all geocoded tweets in the world during 2018–2019 found “a significant relationship between organization on social media and public doubts of vaccine safety” (Wilson and Wiysonge 2020, 5). They argue that it is precisely concerns about risk, regardless of how likely these hazards are to be realized, that give anti-vaccination positions such traction on social media. That is, misinformation can be circulated widely “not because it is considered credible but because, on the unlikely chance it is correct, the consequences would be horrific. More extreme propaganda of negative effects is incentivized, thus leading to a spiral of threat matched by public fear” (Wilson and Wiysonge 2020, 2). This finding evokes François Ewald’s (2002) theory of precaution: if prevention is predicated on trust in science, in knowledge, and the possibility of controlling risks by knowing enough about them to prevent them, a precautionary logic focuses on uncertainty:

Before any action, I must not only ask myself what I need to know and what I need to master, but also what I do not know, what I dread or suspect. I must, out of precaution, imagine the worst possible, the consequence that an infinitely deceptive malicious demon could have slipped into the folds of an apparently innocent enterprise. (Ewald 2002, 286)

A precautionary principle asks that one constantly focus on potential, uncertain dangers. And since one of the many things about which we are uncertain is future danger, the precautionary principle urges the consideration of the *longue durée*, of what could possibly happen years from now. It also creates a new relationship with science, in which the emphasis is less on what is known than what is unknown: it “invites one to anticipate what one does not yet know, to take into account doubtful hypotheses and simple suspicions. It invites one to take the most far-fetched forecasts seriously” (288). As such, a focus on precaution opens an individual or community to the wildest probabilities, most marginal perspectives, and conspiracy theories. Science becomes less the producer of knowledge than the producer of doubts. As Ewald writes, “Prevention is a matter for experts who are confident in their knowledge. Precaution, such as we are seeing emerge today, focuses by contrast on uncertainty—the uncertainty of scientific knowledge itself” (293). Decisions are made in a context of hesitation, anxiety, fear, and mistrust. There is, for Ewald, the dread of “a risk beyond risk, of which we do not have, nor cannot have, the knowledge or the measure” (294).

## Data and methods

The data used in our study comes from the Twitter NZ Vaccine 2021 corpus, which consists of 4,701 tweets using the hashtag #Covid19NZ and its variants (e.g., #covid19nz), and containing the lemmas vaccine* (e.g., *vaccinate*, *vaccines*, *vaccinated*, *vaccination*, etc., as well as related hashtags such as *#vaccinemandate*, *#vaccine_passes*, *#vaccinecertificate*) and vax* (*vaxxed*, *vaxxing*, *vaxxers,* and hashtags such as *#vaxothon*, *#vaxthenation*). We collected these tweets using Python code and the Twitter API. We then used AntConc concordancing software (Anthony 2022) to construct a wordlist and to calculate keyness scores in relation to an existing Twitter reference corpus (see details in the section “Keywords in the Twitter NZ Vaccine 2021 Corpus” below).

To keep the dataset manageable, we also manually inspected the smaller sub-corpus of 1,245. These tweets were randomly selected from within set date ranges and guided by key events, such as changes in vaccination policy, as these events generated larger numbers of tweets using the hashtag #Covid19nz. These 1,245 tweets were manually coded (by one of the authors, with any difficult examples discussed as a team) and analyzed for various linguistics characteristics (of which only the keyness score, see below, and the word category were informative here), as well as their stance toward government measures, theme, mentions of symptoms, and depictions of risk. In accordance with the authors’ previous work on COVID-19 Twitter data (Burnette and Calude 2022; Burnette and Long 2023, there were three categories of clear stance coded for: “supportive of current government measures,” “opposed to current government measures,” and “supportive of government measures but calling for stronger measures.” In addition, instances of neutral stance, for example, tweet announcements of case numbers without additional comment, were coded as “neutral,” and tweets where stance could not be clearly determined were coded as “unclear stance.” The 1,245 coded tweets were produced by 509 unique tweeters, some of whom contributed only one tweet, while, at the other end of the spectrum, one user contributed as many as 85 (this was an outlier as it was the official @NZCovidLive Twitter feed). On average, most users contributed two to three tweets to the sub-corpus, but a great majority of users contributed only one tweet (70%). We have anonymized the tweets cited for publication.

This study employs a mixed methodology that capitalizes on the research team’s varied disciplinary backgrounds. This blend enabled the researchers to combine the larger data set and statistical insights of quantitative analysis with the detailed consideration of relationships, implications, and ambiguities that qualitative analysis frequently unpacks. The initial coding process drew on aspects of thematic analysis (Braun and Clarke 2006; Joffe 2011) and discourse analysis (Paltridge 2021), with close attention given to multimodal features such as emojis, links, and images, as well as lexical decisions and semantic prosody. The subsequent thematic and content analysis (Prior 2014; Neuendorf 2017; Selvi 2019) of the tweets concentrated on the theme of risk, specifically the way users perceived risk and navigated risk prevention strategies, with analysis sections using manifest themes to uncover latent content regarding risk and representations of expertise; risk and appeals to emotion and community; risk and futurity; and risk and modes of risk calculation. The analysis also drew strongly on approaches from English studies, and although the naming of a method and the inclusion of a methodology section is not a norm within the discipline (Felski 2008; Carter 2014), the mode of interpretation commonly used there, and employed in the discussion sections of this article, can be understood as textual analysis. Textual analysis is a method that, like thematic analysis and content analysis, involves close engagement with the text, both its surface and its embedded meanings. It is a multi-layered methodology that responds, as needed, to the content, themes, discursive positions, and assumptions of a text (Belsey 2005). It incorporates a focus on argument, ideology, and logic, gleaned from attention to elements including, but not limited to, language choice, connotation and denotation, syntax, style, and metaphors (Hébert 2022). Textual analysis does not try to uncover the objective truth of the material under analysis but attempts to examine, by unpacking a diverse range of textual attributes, from semantics to cultural origins, rhetoric to ideology, what the text can mean at a particular moment in time, within a particular context (McKee 2003; Kusch 2016).

## Keywords in the twitter NZ vaccine 2021 *Corpus*

We also explored our corpus by extracting keywords (Baker 2010) to gauge the general makeup of the data (Xia, Chen, and Lu 2022). Keyword analysis is a corpus linguistics technique that provides insight into what a given text (or set of texts) is about. This “aboutness” of discourse is not straightforward to glean without computational tools, so keyword analysis constitutes a useful tool to draw statistically significant comparisons between distributions of words in a corpus by comparing them against distributions of words in another (usually larger and more balanced) corpus, called the reference corpus. We performed a keyword analysis using AntConc and a previously collected Twitter corpus containing (largely) New Zealand English tweets (Trye et al. 2019) as a reference corpus. The idea was to extract words that occurred statistically significantly more often in the Twitter NZ Vaccine 2021 corpus than in the reference Twitter corpus (which was not constrained by topic).

Given the polarizing nature of the topic, we compared the results of our keyword analysis with our coded subset of 1,245 tweets to check if there was any relationship between the user’s stance towards government measures and their use of hashtags, with a special interest in whether hashtag use was correlated with individual users’ stylistic preferences and their stance (pro or against vaccines).

The keyword analysis found 249 distinct words that were used significantly more often than expected in the Twitter Vaccine corpus, which we provide in Table 1. All capital letters were removed to standardize spelling; that is, we treated *Delta* and *delta* as the same word in the analysis. The words are organized by grammatical category, distinguishing between (listed in order of frequency): common nouns, attributive words (which include adjectives like *fully*), hashtags (e.g., *#notovaccinemandates*, *#vaccineswork)*, proper nouns, ambiguous attributes or verbs, and a handful of uncategorized words (titles, function words, and the politeness marker *please*^3^).

**Table 1:** Keyword Word Categories and Frequencies

Word category	Frequency	Percentage
Common noun	114	46%
Attribute	40	16%
Hashtag	37	15%
Proper noun	25	10%
Verb	12	5%
Ambiguous (attribute/verb)	9	4%
Other/Unclear	12	5%
TOTAL	249	100%

Unsurprisingly, the keywords are dominated by common nouns (46% of all keywords), particularly the target words used to collect the tweets—*covid, vaccine, vax,* and *vaccination*—followed by attributes (16% of all keywords), with the most salient being *fully, unvaccinated, vaxxed, pfizer, delta*,^4^ and *anti*. A significant thread referenced risk: the noun *risk(s)* itself but also verbs like *reduce* and *protect*, other nouns like *virus, effects, infection, safety*, and *death*, and attributes like *dangerous, safe*, and *serious*—in other words, language largely associated with hazards (Zinn and Müller 2022). Once we combine these keywords with the knowledge gained from our coding of the subset of 1,245 tweets, however, it is clear that for the 20% of tweets opposed to the government’s measures, the language of risk contains *vaccine*, *vaccinated*, and all its variations, as well words like *freedom, mask, government*, and *dhb* (district health board). The keywords, then, present a shifting scene of risks and solutions, the same words often containing diametrically opposed signification.

## Results

Unlike Yang (2021), who found that the negative emotions associated with a pandemic are propagated further than positive ones, we find that although Twitter users might disagree with aspects of the government’s handling of the pandemic, the majority of tweets in our corpus expressed support of the measures (44%) or asked for stronger measures (13%), thereby bringing the total number of pro-vaccination tweets to 57% (see Table 2). Those who were opposed numbered 253, or 20%. The Twitter picture presents a landscape in which most New Zealanders were willing to be vaccinated, a social media snapshot that is in line with the general take-up of the vaccine—by October 16, 85% of the eligible population had one dose, and 65% were fully vaccinated (Manatū Hauora Ministry of Health 2021). It is important to remember that the tweets discursively inhabiting the world risk society are the decided minority: a troubling, intriguing minority but a minority nonetheless.

**Table 2:** Stance and Discussion of Symptoms

Stance	Vaccine symptoms discussed?		Covid symptoms discussed?		TOTAL STANCE
	YES	NO	YES	NO	
Opposed to current government measures	92	161	12	241	253 (20%)
Supportive of current government measures	16	538	39	515	554 (44%)
Supportive of government measures but calling for stronger measures	1	156	9	148	157 (13%)
Neutral	4	119	7	116	123 (10%)
Unclear	5	153	10	148	158 (13%)
TOTAL	118 (9%)	1127 (91%)	77 (6%)	1168 (94%)	**1245**

In our corpus, the majority of tweets focused on medical themes (44% of the tweets we coded), with policy the next highest category at 20% (see Table 3). As such, during this period, the hashtag #Covid19nz was predominantly used to signal and conjoin medical issues rather than, for example, engage with the economic concerns, coping strategies, or environmental gains of lockdown. Significantly, 59% of the tweets with a medical theme emphasized solutions rather than risks, with 165 (51%) of medical solution tweets specifically presenting the vaccine as a means of ending lockdowns and resolving the health risks of the virus (see Table 4). As such, health was on people’s minds, but the medical situation was predominantly seen as manageable and having recognized mitigation strategies, of which the most prominent was the vaccine. Even when we turn to the 24% of medical-themed tweets that discussed medical threats, 38% of these tweets (49 tweets of the non-vaccine-focused threat tweets) did so to then present a solution.

**Table 3:** Tweet Themes

Tweet theme	Frequency	Percentage
Medical	542	44%
Policy	246	20%
Information	129	10%
Subject	104	8%
Personal	58	5%
Mix	56	4%
Government	40	3%
Jacinda Ardern	38	3%
News	26	2%
Unclear	6	0.5%
TOTAL	1245	100%

**Table 4:** Medical Tweet Sub-Themes

Medical tweet sub-theme	Medical issue discussed	Frequency	Sub-theme Totals	Percentage (of medical tweets)
Solution	vaccine	165	322	59%
	other (non-vaccine)	157		
Threat	vaccine	49	129	24%
	other (non-vaccine)	80		
Criticism	vaccine	35	83	15%
	other (non-vaccine)	48		
Unclear		8	8	1%
Totals		542	542	100%

In the midst of this solution-driven health discourse, however, comes the narrative of vaccine danger. Forty-nine tweets, that is, 38% of those who engaged with medical threats, presented the vaccine itself as the threat, which means that as soon as the rhetoric turned to threat (see Medical tweet sub-theme “threat” in Table 4), equal numbers discussed solutions to medical threats (49 of the 80 tweets in the “other” category within the same subtheme) as presented the vaccine as the threat itself. Similarly, when we look at the third focus within medical tweets—criticism at 15%—42%, that is, 35 tweets criticize the vaccine. Significantly, however, across all 1,245 tweets, there were very few tweets—only 6%—that described or engaged with the symptoms of the virus itself (see Table 2). This is, perhaps, due to the low numbers of community cases in Aotearoa New Zealand at this time, but it reveals that even those who were opposed to government measures, which means being opposed to lockdowns, vaccines, and vaccine mandates, did not attempt to downplay the severity of the pandemic by depicting COVID symptoms as mild or harmless.^5^ Instead of dismissing the virus as non-threatening, their rhetorical weight was focused on depicting the vaccination as a grave risk, as those who were against the government’s measures discussed potential medical symptoms of vaccination in 36% of their tweets.

Across our subset corpus, those who were opposed to the measures the government was taking regarding vaccination were the most likely to discuss the symptoms of vaccination, while only 2% of those who were for and strongly for the measures discussed vaccination symptoms (see Table 2). Correspondingly, the larger corpus of Twitter posts we examined contains mostly negative adjective collocates of the lemma vaccine*: words such as *experimental*, *mandatory*, *hospitalised*, and *pseudo*. Given the overwhelming pro-vaccination posts in the corpus, this tells us that those who were pro-vaccination did not tend to use adjectives to describe the vaccine. Their language use suggests that, for them, the vaccine was simply the vaccine, that is, a basic good that did not require modification or adjectival amplification: to refer to it as the “effective vaccine” or the “safe vaccine” was a tautology more likely to undermine than reinforce perceptions of its protective qualities.

### Experts and conspiracies

A risk society is frequently quite prosperous, with a population that is generally well-educated and strives to be actively informed. Yet, the complexity of such societies and their hazards means that it frequently falls to organized power to determine the “reality” of risk, with the result that “affected parties are becoming *incompetent* in matters of their own affliction” (Beck 1992, 53). The entangled global pandemic resulted in COVID policy changes around masking, the need for multiple vaccinations and boosters, elimination, and vaccination mandates, which some users in our corpus struggled with. These users perceived such changes as connected to a sinister desire to control, or they took advantage of the need for policy adjustment as a way to criticize the government. This is clear in the following tweets:

@jacindaardern remember when you and Bloomfield said masks don’t work last year [three laughing emojis] remember when you said vaccinations wouldn’t be mandatory? State of it, hang up the boots love. (User 1)

I’m old enough to remember when you promised the first vaccine would get us back to normal. (User 2)

For Beck (1992), when the public hears about news of things like toxic substances in food, it is a “double shock,” as they are shocked by the event and by the reminder of their loss of sovereignty. Much of the information available to them is, or feels as though it is, hidden behind arcane, sinister processes. This is seen in numerous anti-government tweets in our corpus, exemplified by Users 3 and 4a below, whose tweets question the motivation behind vaccinations by suggesting the interference of sinister conspirators. Or the longer tweet from User 4b, which implies conspiracy without naming an organization:

Have #NewZealand/ers gone stark raving bonkers? Do #Kiwis only read #BigPharma-fed Govt & Media fearmongering disinfo? Even vax-mandating US Govt admitted the vax makes not a jot of difference to transmitting/contracting virus—scientists, too. #COVID19nz. (User 3a)

Trust[ing] Big Pharma is the 2021 mantra. (User 4a)

This mask wearing, distancing & forced v@xing are NOT SAFETY MEASURES. If they really cared about our safety they’d launch a natural immunity campaign. But they don’t want to know anything about such things which proves their real motivation. (User 4b)

By comparison, in the group of those in support of government measures, there was only one tweet that hinted at hidden motivations, and it did so within the context of Indigenous vaccine hesitancy arising from a settler colonial history of racist medical provision:

Currently 57% of people with #Covid19NZ are Māori [Indigenous people of Aotearoa New Zealand]. A suspicious person may wonder if TPU and allied Groundswell NZ are pushing antivax agenda to cause deliberate harm to Māori. (User 5a)

For Beck, in response to an intuitional control of, and seeming denial of, risk, people have had to become private, alternative experts on the risks of modernization (Beck 1992, 61). The complexities of a world risk society have led to an abundance of both supposed experts and spin-doctors, many of whom appear to assess risk at a global, temporal, or technical level remote from individual concerns. Educated populations—and many of those in the global north who hold antivaccination positions are educated—who are skeptical of the government or the medical institution but invested in making informed decisions have to navigate between competing, often politicized claims (Voyles 2020; Halafoff et al. 2022). In the time of social media, the proliferation of polarized opinions and misinformation further complicates the ability to make informed decisions, while simultaneously generating “evidence” for factual and incorrect claims alike. Within our 1,245-tweet subset of coded data, 472 tweets contained sources or other “evidence” to support their claims, using strategies such as linking to videos or articles supporting their position, quoting or retweeting various authorities or experts, or deferring to statistics. In keeping with the “unbounded,” transnational flow of the world risk society (Beck 2002), much of the “evidence” provided by those opposed to government measures stemmed from international sources.

**Table 5:** Stance and Evidence

Stance	Type of Source	Frequency	Percentage	Total Frequency of Tweets Using Evidence
Opposed to current government measures	Government Medical Source	6	4%	138/253 (55% of tweets in category)
	Other Medical Source	17	12%	
	Politician/Policy	5	4%	
	News	40	29%	
	Social Media User	39	28%	
	Other	23	17%	
	Unclear	8	6%	
Supportive of current government measures	Government Medical Source	90	46%	194/554 (35% of tweets in category)
	Other Medical Source	7	4%	
	Politician/Policy	8	4%	
	News	45	23%	
	Social Media User	14	7%	
	Other	7	4%	
	Unclear	23	12%	
Supportive of government measures but calling for stronger measures	Government Medical Source	9	18%	49/157 (31% of tweets in category)
	Other Medical Source	4	8%	
	Politician/Policy	0	0%	
	News	26	53%	
	Social Media User	2	4%	
	Other	3	6%	
	Unclear	5	10%	

Our coding showed that both those who were in support of and those opposed to government measures drew strongly on sources to back up their points. Many, especially those who were pro-vaccine, supported their claims with reference to data provided by government medical authorities such as the Ministry of Health, but their tweets also referred to newspaper articles, local and international, as well as references to other social media users on various platforms, amongst others (see Table 5). It was, however, and in line with estimations of world risk society concerns, users expressing opposition to vaccination who seemed the most concerned with proving their statements: 55% of their tweets included “evidence” from the most diverse range of source types to support their claims, and they were often locked in adversarial engagement with government sources and mainstream media publications.

### Emotional appeals and vulnerable groups

**Fig. 1:**
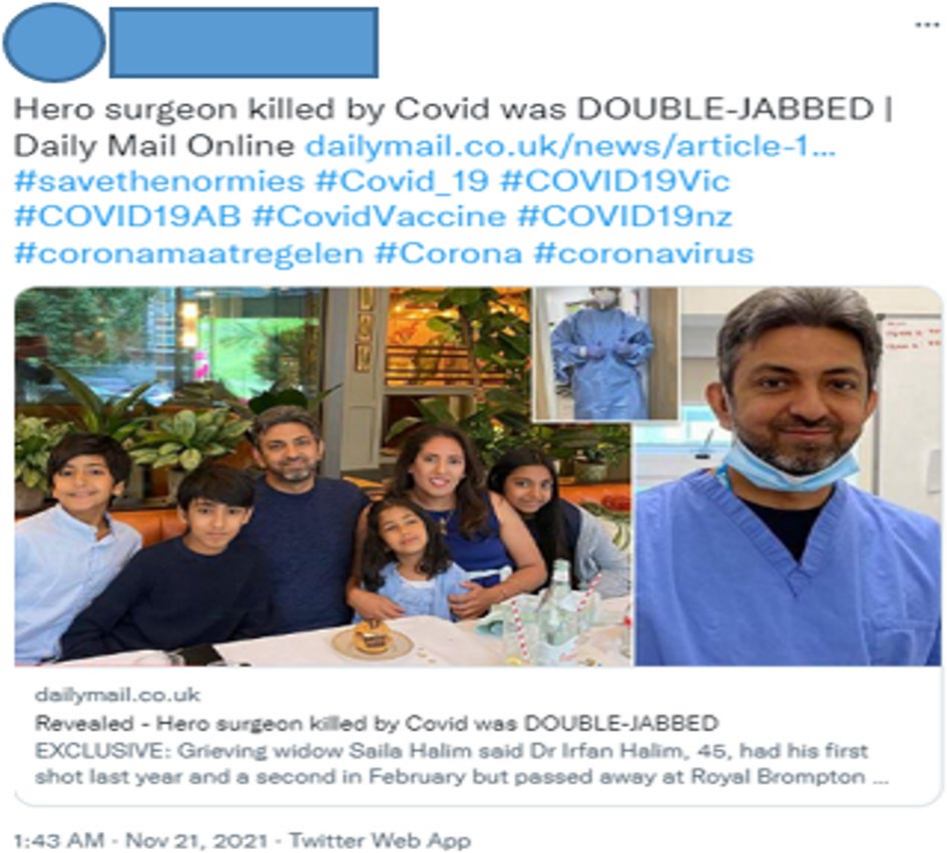
Twitter User 6a

Beck emphasizes the disjoint between scientific objectivity and human connectivity when, writing about climate change and governmental inaction, he insists that events that can be dismissed by scientific communities as anomalies or of minor impact can radically change people’s lives, homes, and children: “‘side effects’ have *voices, faces, eyes* and *tears*” (Beck 1992, 61; italics in original). Beck’s reminder of the humanity of individuals and groups deemed non-statistically significant is important, particularly given the way older members of the population were deemed collateral in many countries during the COVID pandemic (Cook et al. 2021), but it has particular significance for those opposed to vaccination, who regularly presented emotional appeals and phrases in our corpus, and occasionally drew on images to present their position as protecting the groups abandoned by government policies. A tweet by User 6a, shown in Fig. 1, contains a link to a Daily Mail article about Irfan Halim, a surgeon in Australia who died from COVID-19 despite having had two vaccinations. Although the article does not deny the seriousness of the virus, it does cast doubt on the efficacy of the vaccine. The re-post involves three pictures of Halim, two as a surgeon in the hospital where he worked and one as a father out with his family. This juxtaposition—a surgeon who saved lives and a father who created lives—with the user’s description of Halim as having died despite, with this in capital letters, his being “DOUBLE-JABBED,” makes an emotional commentary on the vaccine’s potential impact on families, something capitalized on by anti-government tweeters seeking to influence the risk perception of others.

For Salmon and colleagues, who analyzed antivaccination positions on childhood vaccinations,

the compulsory nature of vaccines for children, the inability of parents to control the risks of adverse reactions, the manmade nature of vaccines, and the unpredictability of adverse reactions, which are dreaded and seemingly exotic, result in parents perceiving the risks of vaccines to be greater than they actually are. (Salmon et al. 2015, 392)

Research by Baker and Walsh (2022) saw this trend continuing during COVID, and we also found that fears of the risks the vaccine presented for children, mothers, and babies were especially prominent in tweets expressing anti-vaccine sentiment. These tweets frequently emphasized the fact that the vaccine was “under clinical trial” and its “long-term safety” was unconfirmed, incorporating emotive pleas, as User 7a did, to “protect mothers and babies.” This plea was also a deeply gendered one, as tweets calling for the protection of children frequently presumed that mothers would take the main caring role and be responsible for decisions around vaccinations. The unknown risk of the vaccine is consistently described in these tweets as more threatening than the perceived short-term, known risk of contracting COVID-19:

it is truly incredible that governments are coercing young and healthy people to get an injection they don’t need that might actually kill them. (User 8)

Or, as insisted by another user, who gives a good example of the collocates found in our keywords, the “newness” of the “experimental gene-based pseudo-vaccine” (User 9a) increases its perceived risk—especially for children. For those opposed to vaccination, the risk posed to children by vaccination is described as so great that “there’s no excuse” for it to be done (User 6b).

Yet, for all the emotional appeals contained in anti-governmental measures tweets, those who were pro-vaccination tended to think in terms of the wider community and use a rhetoric of group care and social defense more regularly than those who were against vaccination. Those for government measures also more frequently incorporated te reo Māori, the language of the Indigenous people of Aotearoa New Zealand, as we see in these tweets:

Please please reach out to your whanau [family] and friends. Kōrero [discuss], explore fears, problem solve, reason and debate. (User 10)

PLEASE Get your whanau and friends vaccinated! And stop breaking lockdown rules! (User 11)

Not including proper names of individuals or places whose official, commonly used name is in te reo, 124, or 22%, of the tweets that were supportive of government initiatives, and 12, or 8%, of the tweets by those calling for stronger measures used te reo words and phrases. Only 6, or 2%, of the tweets by those opposed to the government measures contained te reo.^6^

A narrative of family and care continues in the tweets that call for stronger measures, as the lack of a vaccine for children was a repeated source of concern. As schools were reopened, users worried for the safety of the young—“our most vulnerable population that is unable to be vaccinated” (User 12). Both those for vaccination and against it, then, drew on the rhetorical power of appeals to family and community, but as those opposed to vaccination needed the public not to act, that is, not to get the vaccine, they concentrated on a narrative of risk. As those promoting vaccinations needed the public to act, that is, to be vaccinated, they concentrated on narratives of solution. Hence, as we noted above, the majority of tweets (59%) take a solution-based approach to the pandemic’s medical issues, with the vaccine being the dominant solution (see Table 4).

Similarly, the overall tone of tweets by those in support of government measures is very different from those tweeting against the measures: those in support of vaccinations and lockdowns generally emphasize encouragement rather than harm (Diekema 2022). In so doing, they mirror the rhetoric of kindness and compassion encouraged by the government (Craig 2021). They are informed by and, as we have noted above, cite experts to prove their points but do not demonstrate the sense of doubt and disempowerment seen in anti-government tweets. Particularly during events such as Super Saturday, the Nationwide vaccination drive that saw 130,000 people vaccinated in a day, there was an outpouring of encouragement, mutual reinforcement, and praise (Manatū Hauora Ministry of Health 2021). This tweet is typical of the tone many users adopted:

To the 130K (give or take) people that just got vaccinated today … Thank you. Thank you. Thank you. Thank you. I reckon you all might’ve just helped save Summer. (User 13)

A little earlier, in September, praise was repeated for the mobile vaccination units that were given the particularly Kiwi name of “Shot Bro.” Others encouraged fellow Twitter users to sign up for the vaccine pass, effectively, a certificate of vaccination, by writing that “adding your vaccine pass to your wallet on iPhone is simple as” (User 14). To those who were pro-vaccination, the vaccine mandate was, on the whole, unremarkable—a rule requiring them to do what they intended to do anyway and then to carry a pass that certified that they had done so. As such, the main group of tweeters discussing the vaccine mandate were those opposed to it: 80% of tweets mentioning the mandate did so negatively. For this group, the vaccine mandate carried a strong burden of risk, both to health and sovereignty.

### Temporality and risk

One important dimension of the precautionary valance of the risk society is the fact that the “centre of risk consciousness lies not in the present, but *in the future*. In the risk society, the past loses the power to determine the present. Its place is taken by the future, thus, something non-existent, invented, fictive as the ‘cause’ of current experience and action” (Beck 1992, 34). A vaccine is an act of insurance against a future threat; it tries to protect us from what is to come and is, as such, fundamentally future-oriented. Antivaccination positions are a blurring of present and future risk responses, as the vaccine is both an immediate and future hazard. In Aotearoa New Zealand the SARS-CoV-2 virus was, before the Omicron outbreak, a real and an unreal threat that was simultaneously immediate and remote. The viral threat was real and present in that by the time Aotearoa New Zealand got the vaccine, the country had experienced lockdowns, cases at the borders, and some in the community. At the same time, the viral threat was unreal and remote because the case numbers in Aotearoa New Zealand were extremely low compared to other countries, and as such, the virus frequently seemed to be happening to other people in other places. If the rest of the world was having a pandemic, Aotearoa New Zealand, it often seemed, was having isolation. Compared, then, to the invisible, often remote threat of the virus, for those with reservations, the vaccine was a clear and present danger that caused heart issues (User 7b), reduced T cell immunity (User 9b), stillbirths (User 9c), “viral mutants” (User 16), death (User 17), and even COVID itself (User 18).

The needle in the arm, the jab, has always presented an arresting visual image, and governmental mandates meant that it required an active response in the present: it was a present hazard. At the same time, the threats that are mentioned in our tweets frequently refer to future impacts and long-term effects, which makes the vaccine a risk yet to be realized:

We all need to prepare for some very dark times with mass deaths and ill-health over the next two to three years. And it won’t be the filthy “unvaxed…” (User 19)

**Fig. 2:**
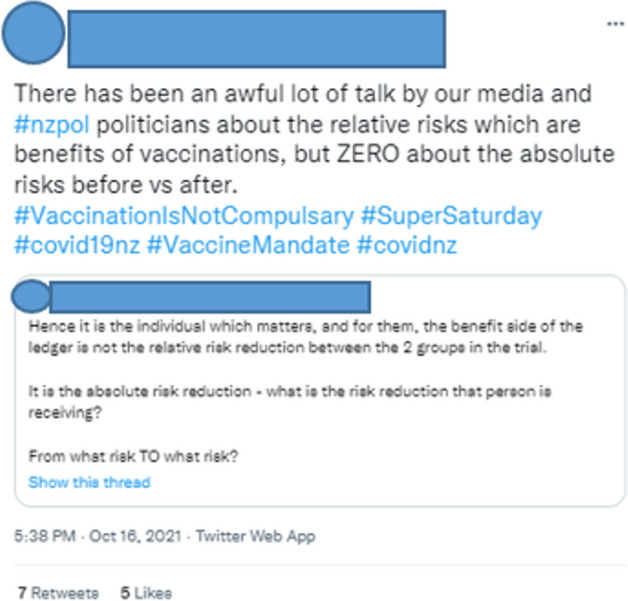
Twitter User 15

Similarly, the user in Fig. 2 argues that a clear distinction between health before and health after the vaccine is more important than attempting to weigh the relative risks and benefits of the vaccine versus the virus. For these Twitter users, the vaccine was a shot in the present that could lead to disaster in the future.

This thinking also led to tweets that criticized the impact of the vaccine mandate on health services, emergency services, and education:

New Zealand can ill-afford to let thousands of firefighters walk off the job because of a pointless vaccine mandate. How dare the government do this (User 20).

My mother is about to be terminated from her job in community mental health. She poses no more of a risk in terms of transmission than a vaccinated person. Tell me how that makes any sense. Also… Don’t we have a mental health crisis? (User 21).

In these tweets, the vaccination mandate was a policy in the present that could lead to social disaster in the future.

These users’ sense of entangled, concealed threat is brought into sharp relief when contrasted with the future thinking in the tweets by those in support of the measures. Although cautious about the impacts of the virus and the potential damage caused by those who didn’t get vaccinated, their emphasis was frequently focused on the future rewards of vaccination rather than future risks, even those of the virus. They encouraged users to get vaccinated so that the country could enjoy Christmas and the summer:

I don’t want anyone to get seriously sick with #Covid19NZ. I don’t want people of Aotearoa to be locked up forever either. Double vaxxing, masking and monitoring our health will help many people feel confident about having a happy summer even if Covid comes our way! (User 5b)

R is nearly 1! Awesome news! Vaccination is working. #rollupyoursleeves to get to enjoy your summer and Christmas! (User 22)

**Table 6:**
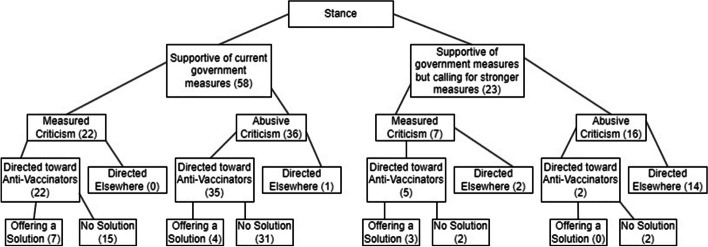
Criticism in tweets by those supportive of government measures and those calling for even stricter measures

However, if the threat of the virus could be met with vaccines and distancing measures, for those in support of the government, or desirous of stricter measures, a more unpredictable risk was other people. As the discussions about border opening began, users expressed concern about the dangers associated with greater movement, as opening up could expose New Zealanders to new variants or to “foreigners who have received low efficiency vaccines” (User 23). Much of the risk perception for these groups, however, was specifically directed at those opposed to vaccination. Although, as noted, the tone of tweets by those supportive of government measures was mainly encouraging, these users often refigured the concerns caused by the pandemic to present anti-vaccinators as an immediate threat. In the few instances in which pro-vaccination tweets expressed hostility through expletives or name-calling, this was directed at those opposed to vaccination 71% of the time (see Table 6). Of the tweets that expressed criticism in a more measured fashion, almost all were directed at anti-vaccinators. The risk caused by the virus was usually addressed by presenting solutions such as vaccines, isolation, and masking, but the risk caused by those who refused to get vaccinated was frequently presented as lacking a clear solution. Only 22% (14 tweets) of the tweets criticizing the threat caused by those opposed to vaccination considered potential solutions—and this is if we include tweets that offer threats like unemployment as a resolution to the anti-vaccinator problem, as well as extremely aggressive tweets that consider the death of anti-vaccinators or the withholding of medical assistance as a “solution.” The group subject to this level of hostility does not always include the vaccine-hesitant: the vaccine-hesitant are usually figured as those who should be helped and guided. But tied up in the risk assessment by those in support of government measures is the creation of a group of committed, inflexible anti-vaccinators, who are often referred to with vitriol. The following tweet is typical of such an approach:

Delta is just getting started and we’ve already gone from 2 deaths in a year to a death every 2-3 days …. Thinking of the non-compliant morons and anti-vaxxers out there who have already killed people … and many more to die. (User 24)

By displacing the threat from an invisible, inhuman virus to a human group presented as unreasonable but knowable, the fear generated by the pandemic is given human form: the anti-vaxxer. In attempting to reduce the size of the unvaccinated population, the community is given a human enemy to vilify and to beat.

### Calculating vaccine risks

Significantly for our considerations of risk, the risks of action are often perceived to be greater or more troubling than the risks of inaction; that is, there is a preference for managing the “adverse health outcomes due to disease after not vaccinating rather than due to vaccinating” (Salmon et al. 2015, 392). If people fall sick from an illness, nature is at fault, but if people fall sick from a vaccine, the perceived fault lies with science and with themselves for agreeing to a medical intervention. There were certainly repeated tweets throughout our corpus that attempted to stress greater risk for those vaccinated:

how much greater the risk is for #vaxxed than #unvaxxed with regard to Delta. (User 3b)

Only one person in our household has had the covid vaccine and they are the only one sick af atm [as fuck at the moment]. (User 25)

These kinds of tweets introduce a confusion that recurs throughout the different stances within our corpus: the threat the unvaccinated can cause the vaccinated. Although the need for widespread vaccination and the potential dangers caused by those who are unvaccinated is a theme among pro-vaccine tweets, the threat of gaps in vaccination uptake is used repeatedly by anti-vaccinators to point to the perceived inadequacy of the vaccine:

What’s the point in a vaccine if it doesn’t protect people from the unvaccinated as per the labour government states? the only thing that protects the unvaccinated is to remove all rights from the unvaccinated? what’s the point in the jab then?????? (User 26)

For Christopher L. Cummings, Shreya Gopi, and Sonny Rosenthal (2021), the decision to engage a primary threat—the disease—because the risks of protective behavior—the vaccination—is perceived as greater is a form of what could be called secondary risk behavior. We see this promotion of secondary risk behavior repeatedly in our corpus.

**Fig. 3:**
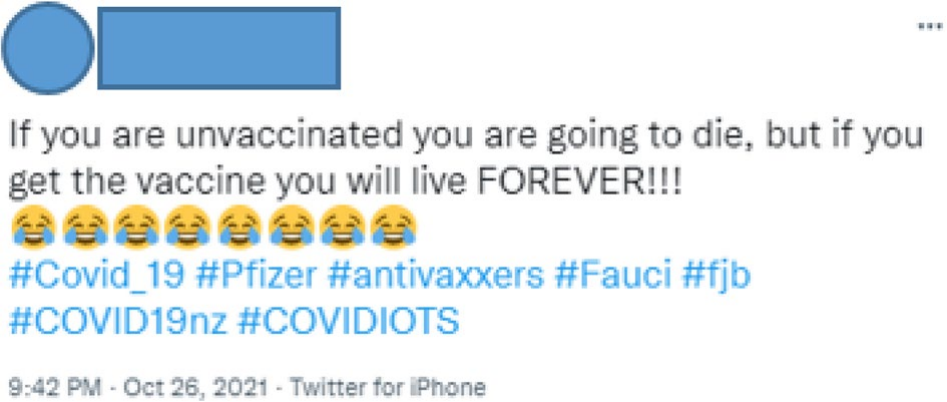
Twitter User 2*7*

A mocking tweet from User 27 in Fig. 3 sheds some light on the disordered mapping of risk we see here. User 27 is clearly deriding those who think the vaccine will provide protection, but their comment on the tendency for narratives of acquired immunity to imply fuller defense than is the case rings true. The encouraging narratives of those who are pro-vaccine in our corpus often imply that vaccinations and compliance with lockdown and masking rules will counter all viral risks. But the fact that the vaccine reduced the severity of the disease, decreased the likelihood of Long COVID, and provided extra protection against transmission rather than providing absolute immunity was well known by this period in 2021 (see, for example, Stuff 2023; Haas et al. 2021). The reference to government experts and links to academic and press reports in our tweets makes it unlikely that the tone of encouragement found in the pro-government tweets stemmed from a lack of knowledge about the virus or the vaccine’s benefits. Instead, User 27’s tweet speaks more to the risk assessment issues by those who were vaccine-hesitant and committedly anti-vaccine. That is, the faulty mapping of risk we’ve seen above—if the vaccine is so good, why are the vaccinated catching COVID?—depends on the idea that the vaccine will provide complete immunity to the virus.

If the most extreme anti-vaccine tweets, the ones that complied with so many positions and phrases stereotypically associated with anti-vaccinators, were often from the same, smaller number of accounts, the tweets that seemed driven by a less aggressive, overt agenda were often by those who simply asked what the point of a vaccine was if it didn’t give full protection:

if you have the vaccine you are supposed to be protected yet you can still catch it[.] unvaccinated people don’t magically make the virus (User 28).

The confusion expressed by some tweeters might be genuine, but for those wishing to deliberately sow non-compliance with vaccine policies, these kinds of posts could be used to propagate poor risk assessments. They confound risk management by implying that if the vaccine is not completely effective at preventing transmission, then it should be abandoned, as its possible side effects loom larger in rhetorical value. The unknown, unquantifiable threat in the future is presented as a greater risk than the known, if unpredictable, reasonably quantifiable threat in the present. For Megan Brown’s pre-pandemic research, these kinds of deliberations create “redundant risk,” that is, situations in which all courses of action seem potentially hazardous (Brown 2011, 143). Faced with surplus, often unmappable risks, people can become anxious, pessimistic, and incapable of deciding the best course of action, which leads to vaccine hesitancy and anti-vaccine positions.

### Hashtags as strategic markers of disruption

We draw our discussion on risk to a close with a return to the keywords found in our corpus. The keywords were most commonly nouns, but more surprising, however, was the next most significant category of keywords: hashtags. We disregarded the hashtag #Covid19nz and its variations, as its presence was necessarily expected in every tweet, but even with this omission, hashtags were a dominant type of keyword. Findings from our corpus, Twitter NZ Vaccine 2021, suggest that tweeters often employed hashtags in their posts, and it appears that the hashtags chosen were not just used to locate the post in a particular discourse topic or community—that is, functioning for affiliative purposes (Zappavigna 2011)—but also to clarify the user’s message, in other words, for information transmission functions (Wikström 2014). Examples include: *#nzpol, #lockdownz, #getvaccinated, #auspol, #novaccinemandates, #novaccinepassports, #covidnz, #covidvaccine, #resignjacinda*.

The use of hashtags varied across tweets, ranging from one to 32. However, most tweeters who contributed multiple tweets were fairly consistent in the number of hashtags they used across their various tweets. As we were interested in individual preferences and styles in the use of hashtags, we calculated differences between tweets with the largest and smallest number of hashtags to gauge how consistent users were. Our results show that the average difference had a mean of 0.55 and a mode of 0, which points to a remarkable consistency. Many tweeters use only one hashtag in each of their tweets, a great majority of tweeters use two or three in most of their tweets, and very few users include 10 or more hashtags. Putting these two observations together shows that while hashtag use may vary across users (inter-speaker variation), it does not vary across their tweets (intra-speaker variation). Put simply, tweeters either use many hashtags or few hashtags, and their linguistic hashtag patterns tend to remain consistent across posts.

**Fig. 4:**
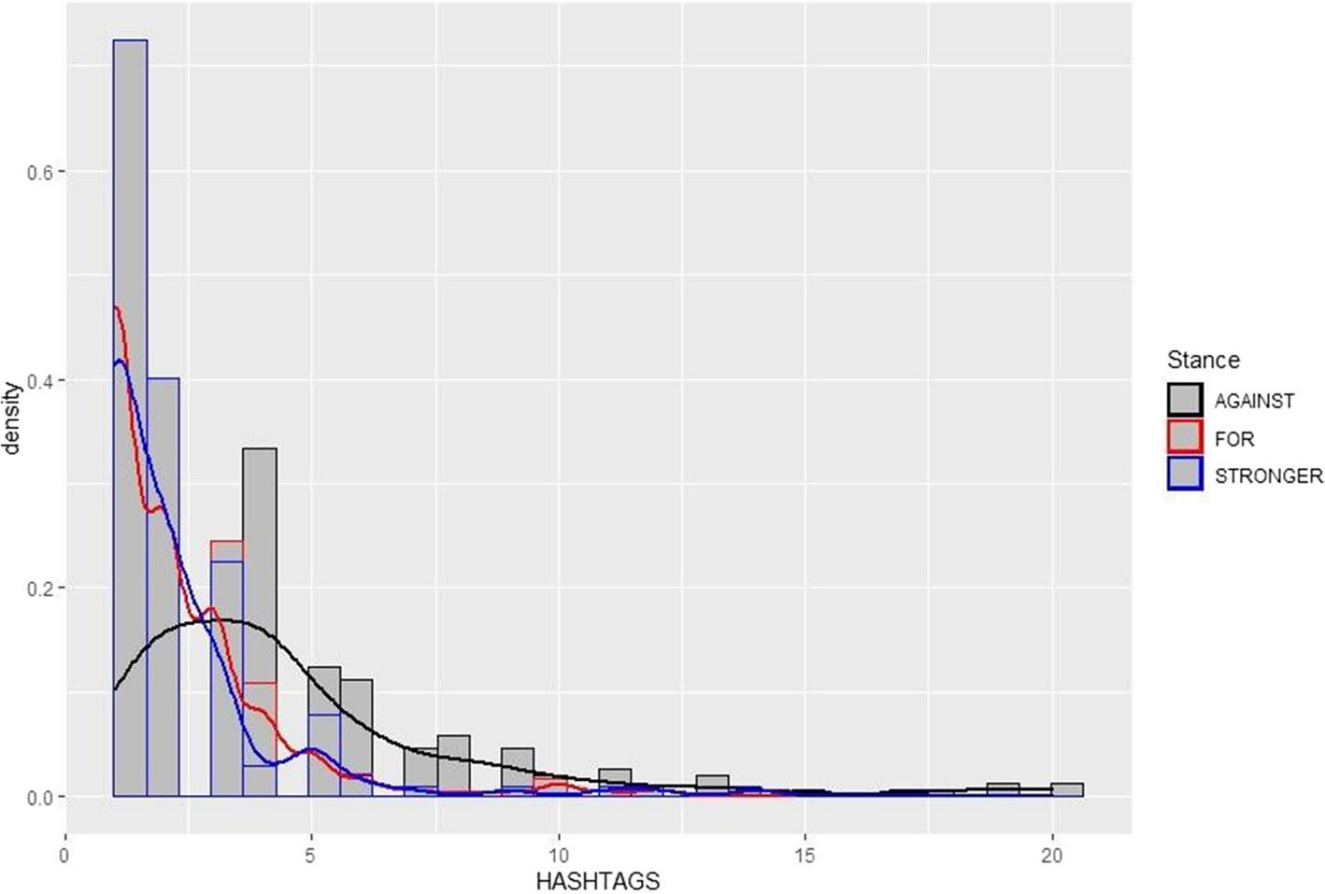
Relationship between number of hashtags and stance (removed one outlier tweet containing 32 hashtags)

The second finding related to the connection between the stance of the tweets (towards government measures; here, largely vaccines and vaccination processes) and the number of hashtags used. We explored the relationship between hashtag use and stance by means of a histogram, together with a density distribution (Fig. 4).

**Fig. 5:**
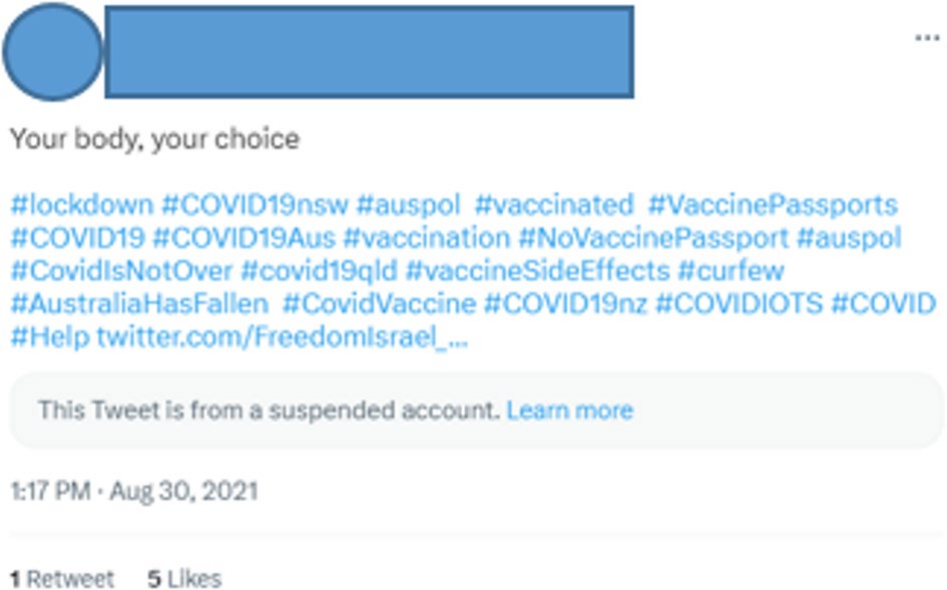
Tweet from Twitter User 29

The figure shows that tweets that are in support of government measures or call for stronger measures tend to contain, on average, fewer hashtags (the red and blue lines), whereas those that are against government measures tend to have more hashtags. In other words, an increase in hashtags correlates with an increase in dissatisfaction with the status quo. Putting both findings together reveals that those who were in favor of government measures consistently used fewer hashtags, while those who were against vaccinations consistently used more. We hypothesize that the searchability and discoverability of hashtags make them particularly useful for users whose aim is to widen their community of reach and to attract more interest to their posts. This allows users to affiliate with like-minded people who are similarly feeling excluded from dominant societal discourses. Somewhat unexpectedly, we discovered several tweeters posting anti-vaccination messages that included pro-vaccination hashtags alongside anti-vaccine ones, as with User 29 in Fig. 5: this may indicate that some users also sought to disrupt or co-opt pro-vaccination discourse.

As we have shown, the tweets rhetorically and ideologically opposed to vaccinations depicted Aotearoa New Zealand as trapped in the hazards of a world risk society. These tweets emphasized threat and ulterior government motives and encouraged a disordered, convoluted mapping of risk. We suggest that the use of numerous hashtags in these instances operates as a performance of non-prescriptive risk amplification: that is, a profusion of hashtags suggests without stipulating entangled connections, causes, and effects. Take, for example, the following:

The year of delivery failed miserably, the year of the vaccine is failing miserably, our only hope now is that the year of the Mandate fails miserably too #nzpol #3waters #covid19nz #vaccinemandate #lockdownnz #ResignJacinda (User 30).

User 30 does not prescribe any specific connection between the hashtags, particularly the link between the government’s vaccine mandates and the Three Waters Reform Programme, which planned to move the management of Aotearoa New Zealand’s drinking water, wastewater, and stormwater to publicly owned service entities, but it is this lack of prescription that is so powerful (Te Tari Taiwhenua Department of Internal Affairs 2023). The lack of specific conjunctions between the hashtags means that readers can connect them as they wish—or, to use the language of conspiracy, by doing their own research and drawing their own conclusions (see, for example, Hughes et al. 2021). The proliferation of hashtags, then, mirrors the paranoid risk mapping of conspiracy theories, implying connections and agendas across departments, institutions, and nations where none might exist. It encourages disordered, risk-focused thinking, implying but not prescribing, leaving space for each reader to navigate and connect the hazards. Risks are thereby escalated without being specified, which is a powerful way to increase a sense of dread.

## Conclusion

Between August and November 2021 in Aotearoa New Zealand, the vaccine was at the heart of the pandemic’s risk nexus, a solution to the pandemic that, for some, was also the pandemic’s primary risk: either by directly causing future health risks or by creating a group of anti-vaxxers against whom the ire and frustration of the majority could be directed. Beck’s world risk society is a modernity of hazards, of tangled, transnational complexity of cause and effect in which risk is increasingly hard to assess, in which governments seem to be responding poorly, and experts too focused on broad or conservative assessments. The insights into risk that Beck’s work affords us indicate the continuing relevance of the concept of a world risk society, not only into the structures and complexities of modern life but also into the attitudes and values the public draws in their risk assessments.

The contextualization of Beck’s work for the Aotearoa New Zealand experience of the pandemic and the insights this paper offers into public perception of pandemic risks, including the vaccine and public take up, can aid future epidemic and pandemic responses as well as vaccination programs. Further research on risk, vaccines, and health emergencies that draws on social media data could employ our research methodology and Beck’s framework to conduct further engagement into the overlap between anti-vaccine positions and climate activism, mental health concerns, or the connections between risk, vaccines, and shame in Aotearoa New Zealand or other countries. As with all research using social media data, however, our study cannot claim complete representativeness—in this instance, of all New Zealanders. Twitter, now X, as a platform skewed toward younger users, is not without bias and may not accurately reflect the perspectives of other demographics. As mentioned in the Results section above, the data in this study has a high percentage of tweets in support of government measures, which contradicts previous findings (Yang 2021). In the future, it would be beneficial to compare the findings of this paper with data from additional platforms to develop a more holistic understanding of New Zealanders’ attitudes toward vaccination during the COVID-19 pandemic.

If the virus catapulted a world suffering a climate emergency further into the entanglements of a world risk society, responses to the vaccination caused even greater escalation. Pre-existing social inequity meant that certain social groups were more at risk in the pandemic than others, and in our data, the tweets seeking stronger measures were particularly concerned about vaccination rates for Māori as well as the unavailability of vaccinations for children during this period. But in addition to the disparities impacting communities, perceptions of risk also determine a group’s place within a world risk society. That is, the value we see in Beck’s work in this paper is not its blanket mapping of the harms impacting all citizens but its use in illuminating the perceptions of risk within the group of those opposed to the vaccine, be they committed anti-vaccinators or vaccine-hesitant. As we have argued, only those who expressed opposition to vaccination were responding to a situation that maps on to Beck’s world risk society. Those who considered the virus a greater threat than the vaccine did not exhibit the same patterns of concern.

Our data reveals a rhetorical pattern positioning those firmly opposed to or hesitant about the vaccine within a pandemic space of entangled medical and political hazards, while those who were pro-vaccine inhabited a pandemic space that was solution-led. For those opposed to vaccination, government experts were to be distrusted, conspiracies abounded, and children were at risk. Their hashtag-abundant tweets encouraged disordered reading and frequently employed a logic of fearful precaution about an unknowable, hazardous future. For those who were pro-vaccination, expertise reassured, science and the government were flawed but basically trustworthy, families could and should be protected, and threat of the virus could be mitigated by lockdowns and vaccination. Their one point of overlap with a world risk society, in which a precautionary logic was employed, was in regard to the threat caused by those who were opposed to vaccinations and government measures. In the final months of 2021, then, it was not the virus that captured Twitter users’ imaginations, it was the vaccine and it was other people.

## Data Availability

The data that support the findings of this study are available from the authorship team upon reasonable request.
